# Perfil de IL-6 e TNF na Formação de Células Espumosas: Um Método Aprimorado Usando a Sonda de Isotiocianato de Fluoresceína (FITC)

**DOI:** 10.36660/abc.20210682

**Published:** 2022-07-28

**Authors:** Cynthia Aparecida Castro, Tereza Cristina Buzinari, Rafael Luis Bressani Lino, Heloisa Sobreiro Selistre de Araújo, Fernanda de Freitas Aníbal, Roberto Mario Machado Verzola, Vanderlei Salvador Bagnato, Natalia Mayumi Inada, Gerson Jhonatan Rodrigues

**Affiliations:** 1 Instituto de Física de São Carlos Universidade de São Paulo São Carlos SP Brasil Instituto de Física de São Carlos, Universidade de São Paulo, São Carlos, SP – Brasil; 2 Departamento de Morfologia e Patologia Universidade Federal de São Carlos São Carlos SP Brasil Departamento de Morfologia e Patologia – Universidade Federal de São Carlos, São Carlos, SP – Brasil; 3 Departamento de Ciências Fisiológicas Universidade Federal de São Carlos São Carlos SP Brasil Departamento de Ciências Fisiológicas – Universidade Federal de São Carlos, São Carlos, SP – Brasil

**Keywords:** Aterosclerose, Inflamação, Células Espumosas, Lipídios, Placa Aterosclerótica, Fatores de Risco, Isotiocianatos, Fluoresceina

## Abstract

**Fundamento:**

A formação de células espumosas ocorre devido ao aumento em lipoproteína plasmática de baixa densidade (LDL) e desregulação da inflamação, sendo importante para o desenvolvimento da aterosclerose.

**Objetivo:**

Avaliar o perfil do fator de necrose tumoral alfa (TNF-α) e da interleucina-6 (IL-6) no método de formação da célula espumosa existente, otimizando esse protocolo.

**Métodos:**

A LDL foi isolada, oxidada e marcada com sonda de isotiocianato de fluoresceína (FITC). As células espumosas foram geradas de célula derivada de monócitos humanos THP-1 e incubadas na ausência (controle) ou presença de FITC-ox-LDL (10, 50, 100, 150 ou 200 μg/mL), por 12, 24, 48 ou 72 horas. A FITC-ox-LDL na célula foi quantificada por microscopia. O ensaio de imunoabsorção enzimática foi avaliado para quantificar a IL-6 e o TNF-α, com um p <0,05 considerado significativo.

**Resultados:**

Todas as concentrações de FITC-ox-LDL testadas apresentaram fluorescência mais alta em comparação com o controle, demonstrando maior acúmulo de lipoproteínas nas células. Quanto mais alta a concentração de FITC-ox-LDL, maior a produção de TNF-α e IL-6. A produção de IL-6 pelas células espumosas foi detectada até o valor de 150 µg/mL da LDL máxima de estímulo. Concentrações acima de 50 μg/mL de LDL estimularam maior liberação de TNF-α comparado ao controle.

**Conclusões:**

Nosso modelo contribui para o entendimento da liberação de IL-6 e TNF-α em resposta a várias concentrações de ox-LDL usando o método otimizado para a formação de células espumosas.

## Introdução

A aterosclerose é uma das causas mais importantes de morbidade e mortalidade em todo o mundo, e é detectada pelo acúmulo de lipídeos em macrófagos que nesse estágio são conhecidos como células espumosas no espaço subendotelial da parede arterial.^1^ A formação de células espumosas ocorre pelo aumento da lipoproteína plasmática de baixa densidade (LDL), que passa por vários processos fisiológicos mediados por oxidação, acetilação e desnaturação. Essas modificações são estímulos fisiológicos que favorecem a internalização de partículas lipídicas por macrófagos, gerando a célula espumosa.^2^ Tipos de células alternativas presentes na neoíntima, tais como músculo liso e células endoteliais, também podem internalizar gotículas de lipídeos e se transdiferenciar a um estado semelhante ao das células espumosas de macrófagos, contribuindo para a formação de placa aterosclerótica.^3 , 4^

Os macrófagos podem contribuir para o desenvolvimento da aterosclerose, exibindo alta heterogeneidade^5^ devido a seu fenótipo resultante. Esse fenótipo pode ser classificado como M1 ou M2. Macrófagos M1 são caracterizados como pró-inflamatórios e têm alta expressão de proteínas pró-inflamatórias que contribuem para a formação da placa aterosclerótica. Os macrófagos M2 desempenham um papel preventivo, reduzindo o tamanho da placa e melhorando sua estabilidade, já que tem um perfil anti-inflamatório.^5 , 6^

Estimular o perfil pró-inflamatório é importante no processo de formação das células espumosas, já que mecanismos inflamatórios podem atuar como precursores na formação lipidocêntrica, além de promoverem a aterogênese por meio da absorção de colesterol e da redução do efluxo de colesterol.^2^ Embora a hiperlipidemia estimule a aterogênese fornecendo mais lipídeos para a formação de células espumosas, alguns mediadores inflamatórios aumentam a oxidação lipídica, tais como o fator de necrose tumoral alfa (TNF-α) e interleucina-6 (IL-6).^7^ A IL-6 é uma citocina pleiotrópica que exibe propriedades pró- e anti-inflamatórias, dependendo do tipo de célula-alvo. O aumento da IL-6 na aterosclerose resulta em efeitos em várias células envolvidas no processamento de lipídeos e na formação de placa, tais como a ativação de células endoteliais, a proliferação celular de músculo liso, e o acúmulo de lipídeos de macrófagos.^8^ Agora existem fortes evidências para o papel do TNF-α derivado de macrófagos no desenvolvimento da aterosclerose e no aumento da inflamação vascular.^9^ Portanto, é útil investigar a fisiopatologia da formação de células espumosas para o desenvolvimento de novas intervenções terapêuticas para a aterosclerose.^10^

As técnicas mais comumente usadas para o estudo da formação da célula espumosa são a quantificação de ox-LDL marcado dentro dos macrófagos ou o uso de corantes não específicos, tais como o Oil Red O. O objetivo deste estudo foi avaliar o perfil do TNF-α e da IL-6 no método de formação da célula espumosa existente, otimizando, assim, esse protocolo. A presença desses mediadores inflamatórios age como marcadores da formação das células espumosas pró-inflamatórias, o início da formação da placa aterosclerótica.

## Materiais e métodos

### Produtos químicos e reagentes

Este estudo utilizou RPMI 1640, soro fetal bovino (SFB) (Vitrocell Embriolife, Campinas, SP, BR), PMSF (fluoreto de fenilmetilsulfonil); forbol 12-miristato 13-acetato (PMA), isotiocianato de fluoresceína (FITC), dicloridrato de 4’, 6-diamidino-2-fenilindol (DAPI), benzamidina, cloranfenicol e gentamicina, aprotinina, brometo de tiazolil azul de tetrazolio (MTT), que foram comprados da Sigma-Aldrich, St. Louis, MO, EUA; kit de ensaio de colesterol Amplex Red (Nº de catálogo A12216, Invitrogen, Molecular Probes, Eugene, OR); IL-6 e TNF-α R&D Systems, 614 McKinley Pl NE, Minneapolis, MN, EUA.

### Isolamento de LDL

O presente estudo foi aprovado pelo Comitê de Ética em Pesquisa Humana da Universidade Federal de São Carlos - UFSCar (nº 2.243.706) e os participantes forneceram seu consentimento por escrito. Foi realizada a coleta de sangue de 10 voluntários normolipidêmicos (homens e mulheres com idades entre 18 e 45 anos) e o plasma foi obtido após a centrifugação a 1000 g por 15 min na presença de K_2_EDTA 0,1 mL por 5 mL de sangue. Depois disso, benzamidina (2 mM), gentamicina (0,5%), cloranfenicol (0,25%), PMSF (fluoreto de fenilmetilsulfonil) (0,5 mM), e aprotinina (5 µl/mL) foram acrescentados ao plasma (todos adquiridos da Sigma-Aldrich, St. Louis, MO, EUA) conforme descrito no relato anterior.^11^ A densidade do plasma foi elevada a 1,021 g/mL por KBr (o volume de plasma é multiplicado pelo fator 0,3265 e o valor é obtido em gramas de KBr sólido). Em seguida, 2,5 mL de plasma foram acrescentados ao tubo de polipropileno (4 mL) e o tubo foi preenchido com solução de KBr d = 1,006. A LDL foi isolada por ultracentrifugação (337 g por 4 horas a 4 °C) em um rotor de ângulo fixo SW60TI (Beckman Coulter, Beckman). A fração amarelo-laranja da LDL permaneceu no infranadante. A fração da LDL foi coletada por sucção, usando-se uma seringa de 1 mL. A LDL coletada foi dialisada em ambiente escuro a 4 °C em 2 L de PBS, pH 7,4, com quatro trocas de PBS por 24 horas. Após a diálise, a LDL foi filtrada (0,22 μm) e armazenada a 4 °C. A concentração de proteína foi determinada usando-se o método do reagente de fenol de Folin.^12^

### Modificação oxidativa da LDL

A LDL oxidada (ox-LDL) foi obtida pela incubação da LDL com CuSO4 (5 μmol/mL por mg de proteína LDL/4 h/37 °C). A oxidação foi interrompida acrescentando-se 20 µmol/mL EDTA. O grau de oxidação foi determinado pela medição da oxidação ferrosa do laranja de xilenol.^13^ Após a oxidação, a ox-LDL foi dialisada em ambiente escuro por 24 h a 4 °C e lavada 4 vezes com 2 L de PBS e EDTA (0,3 mM).

### Marcação fluorescente de LDL

A LDL foi marcada com isotiocianato de fluoresceína (FITC). Todos os procedimentos foram realizados em ambiente escuro. A LDL (1 mg/mL) e o FITC (50 μg /mL) foram misturados e incubados a 37 °C por 3 horas. O FITC não ligado foi retirado por diálise em PBS, por 48h a 4 °C com oito trocas de PBS, e filtrado por uma malha de 0,22.^14^ O FITC-ox-LDL foi armazenado a 4 °C e usado por até dois meses.

### Cultura celular

A linha celular derivada de monócitos humanos THP-1 foi comprada do *Banco de Células do Rio de Janeiro,* RJ, *Brasil,* e foi cultivada a 37 °C em uma atmosfera de 5% CO_2_ com uma densidade de 10^6^ células/mL. O meio de cultura para as células THP-1 foi o meio RPMI 1640 suplementado por soro fetal bovino (SFB) a 10% (Gibco BRL), 50 mg/L de sulfato de gentamicina, e 2 mg/L de anfotericina B. Para os experimentos, foram utilizadas células THP-1 para indução em macrófagos, usando o forbol 12-miristato 13-acetato 100 nM (PMA, Sigma)^15^ e interferon (IFN)-γ (500 U/mL) para o fenótipo M1 induzido.^16^ O PMA induz a diferenciação da célula THP-1 pela interação direta com PKCδ que se liga à trombomodulina, ativa ERK1/2, que, por sua vez, ativa a expressão do inibidor de ciclo da célula p21^Cip1^via sinalização de NF-kB p65. Além disso, ERK1/2 participa da fosforilação de paxilina, cofilina, LIMK1, e PYK2, que mediam a remodelação citoesquelética para promover a diferenciação.^17^ O interferon γ (IFN-γ), pelo ativador de transcrição 1 (STAT1), favorece a polarização de macrófagos M1, que produzem mediadores pró-inflamatórios, incluindo o TNF-α, IL-6, IL-1.^18^ Depois dessa indução, as células de macrófagos THP-1 foram incubadas sem FITC-ox-LDL ou com 10, 50, 100, 150, 200 μg/mL, durante tempos diferentes (12, 24, 48 ou 72 horas) dependendo do propósito do experimento.

### Captação celular de colesterol

Para induzir a diferenciação do monócito THP-1 em macrófagos, monócitos THP-1 (10^4^ células/mL) em placas de 96 poços foram tratados com 100 nM de PMA por 48 horas a 37 °C. Para identificar a melhor concentração de ox-LDL para induzir a formação de célula espumosa, foi realizada uma curva de concentração-resposta: por 24 horas a 10 μg/mL, 50 μg/mL, 100 μg/mL, 150 μg/mL, e 200 μg/mL FITC-ox-LDL + IFN-γ (500 U/mL). Para a análise temporal, células diferenciadas foram incubadas na ausência ou na presença de FITC-ox-LDL (100 μg/mL) + interferon γ (500 U/mL) por 12h, 24h, 48h e 72h. O núcleo celular foi marcado com 1 μg/mL de sonda fluorescente DAPI (Sigma) por 10 minutos e lavado 3 vezes com PBS. Para analisar a imagem de fluorescência, foi usado um sistema de microscópio de fluorescência, ImageXpress Micro (Molecular Devices) com excitação de 495nm e 525 nm de emissão de FITC-ox-LDL, e excitação de 340 nm e 488 nm de emissão de DAPI

### Quantitação de colesterol/éster de colesterol em lisado celular

O teor de éster de colesterol da célula espumosa foi quantificado pelo kit de ensaio de colesterol Amplex Red (Nº de catálogo A12216, Molecular Probes, Eugene, OR), de acordo com o protocolo do fabricante. Para essa análise, as células THP-1 (2x10 células/poço) foram cultivadas em placas de 6 poços, diferenciadas em macrófagos, conforme descrito acima, e incubadas com ou sem ox-LDL. As células espumosas foram fixadas em paraformaldeído a 2% por 15 minutos, lavadas uma vez com PBS e incubadas com 200 μl/poço de álcool absoluto por 30 min a 4 °C para extrair lipídeos celulares. O teor de colesterol foi determinado incubando-se 50 µl de lipídeos extraídos de álcool diluídos em um tampão de reação 1x (0,1 M K_2_HPO_4_, pH 7,4, 0,05 M NaCl, 5 mM de ácido cólico, 0,1% Triton X-100) ou solução não diluída com 50 µl da solução de ensaio (colesterol total) ou 50 µl da solução de ensaio sem a esterase do colesterol (colesterol livre), por 30 min a 37 °C em ambiente escuro, e, em seguida, medindo-se a fluorescência (fluorímetro de microplaca HTS-7000; excitação de 530 nm, 590 nm). O valor foi relativizado para o os níveis de proteína celular totais. Para quantificar os níveis de proteína celular totais, as células espumosas extraídas de lipídeos foram incubadas com 0,1% (razão peso/volume [p/v]) SDS/0,2 M NaOH por 30 min em temperatura ambiente para extrair proteína celular. Os níveis de proteína celular foram determinados usando-se o método do reagente de fenol de Folin.^12^ Os níveis de colesterol total e éster de colesterol foram representados como nanogramas de colesterol total ou éster de colesterol por micrograma de proteína.

### Medições de citocina

A quantificação de citocinas inflamatórias em lisado de célula espumosa foi realizada usando-se o ensaio de imunoabsorção enzimática (ELISA). A concentração de IL-6 e do TNF-α nos sobrenadantes de macrófagos foi medida usando-se o DuoSet kits (R&D Systems, 614 McKinley Pl NE, Minneapolis, MN, EUA). Os macrófagos incubados na ausência de ox-LDL foram definidos como grupo de controle (M).

### Viabilidade celular

A viabilidade celular foi determinada por MTT (brometo de tiazolil azul de tetrazolio) (Sigma- Aldrich, St. Louis, MO, EUA) (Mosmann, 1983). Monócitos de THP-1 (10^4^ células/mL) foram inoculados em placas de 96 poços e tratados com 100 nM PMA para diferenciação de macrófagos, por 48 horas, mantidos a 37 °C em uma incubadora umidificada contendo 5% CO_2_. Após 48 horas, as células foram expostas por 24 horas a 10 μg/mL, 50 μg/mL, 100 μg/mL, 150 μg/mL e 200 μg/mL de FITC-ox-LDL + interferon γ (500 U/mL). A análise da viabilidade celular ao longo do tempo também foi realizada, sendo as células incubadas na ausência (grupo de controle - M) ou na presença de FITC-ox-LDL (FC) (100 μg/mL) + interferon γ (500 U/mL) por 12h, 24h, 48h e 72h. Posteriormente, foram adicionados 5 mg/mL de MTT, seguido de 4 horas de incubação a 5% CO_2,_ 37 °C. Depois desse tempo, foram adicionados 100 μL de dimetilsulfóxido (DMSO) e a placa permaneceu no agitador de placas por 10 minutos. A absorbância foi medida em 540 nm, usando-se um leitor de microplaca SpectraMax GeminiXS (Molecular Devices, Sunnyvale, CA, EUA).

### Análise estatística

Todo o estudo foi realizado pelo menos em triplicata em três experimentos independentes, de acordo com as recomendações para Boas Práticas de Cultura Celular (BPCC).^19 , 20^ A normalidade dos dados foi verificada pelo teste Kolmogorov-Smirnov; igualdade de variância (teste de Levene). Todos os valores são expressos em média ± desvio padrão (DP). Para se determinar a diferença entre condições, foi aplicado o ANOVA com o teste post hoc de Bonferroni para múltiplas comparações. Para de determinar a diferença entre duas condições, o teste T não pareado de Student foi usado (SigmaStat versão 3.5; Systat). O nível de significância adotado para a análise estatística foi 5%.

## Resultados

**Figura 1:**
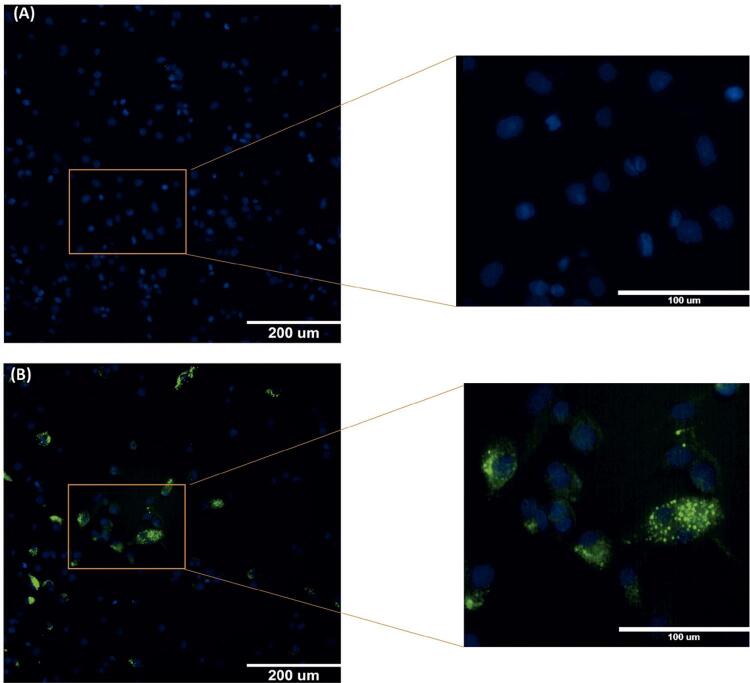
Imagens representativas de captação de FITC-ox-LDL em macrófagos de THP-1. As THP-1 foram incubadas na ausência (grupo de controle - A) ou presença B das concentrações indicadas de FITC-ox-LDL por 24 horas. As células foram então lavadas, fixadas e examinadas usando-se um conjunto de filtro de 546 nm. A captação de FITC-ox-LDL foi mostrada em verde e os núcleos das células foram marcados com DAPI (azul).

Houve um acúmulo maior de ox-LDL marcado com uma sonda de FITC (indicado pela presença de fluorescência verde), não apenas na área perinuclear, mas também distribuído pelo citosol da maioria das células, conforme mostrado na Figura 1B . Como esperado, não foi encontrado acúmulo de LDL em células não tratadas ( Figura 1A ), que apresentou apenas fluorescência azul, que marca o núcleo da célula.

**Figura 2:**
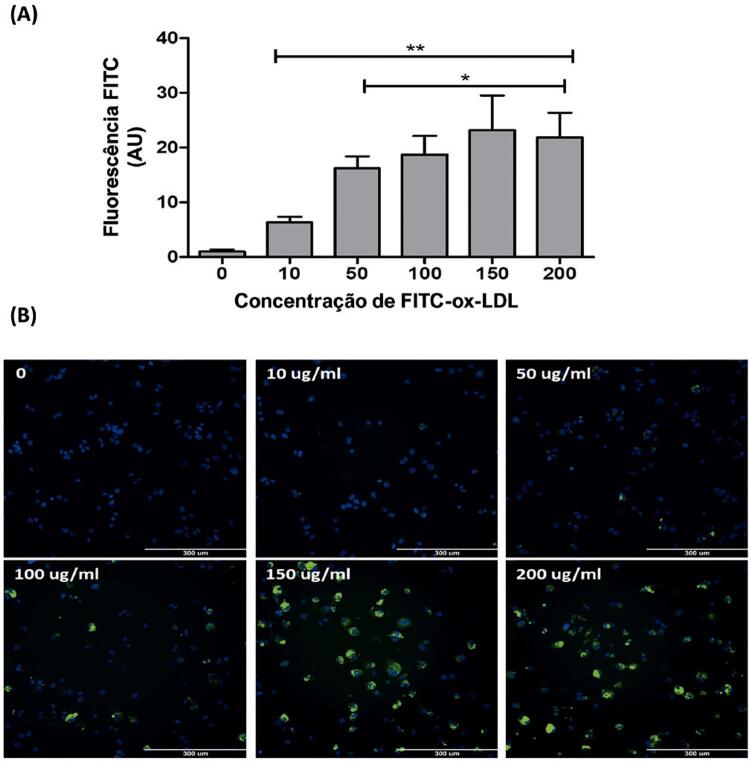
Medição da concentração de FITC-ox-LDL em células THP-1. A) Captação de colesterol fluorescente dependente da concentração por macrófagos THP-1 em unidades arbitrárias (UA). B) Imagens representativas de captação de FITC-ox-LDL em macrófagos de THP-1. As THP-1 foram incubadas na ausência (grupo de controle: 0) ou na presença das concentrações indicadas de FITC-ox-LDL (10 – 200 μg/mL) por 24 h. A captação de FITC-ox-LDL foi mostrada em verde e núcleos celulares foram marcados usando-se DAPI (azul). As células foram visualizadas em microscópio de fluorescência (20× objetivas). Os valores foram expressos como média ± DP. * P <0,05, comparado a células incubadas com 10 μg/mL; ** P <0,01, comparado a células incubadas na ausência de FITC-ox-LDL.

Na figura 2B , as imagens de fluorescência microscópica mostram que a FITC-ox-LDL foi absorvida em todas as concentrações, apresentando fluorescência mais alta quando comparada ao controle, usando-se a incubação de 24 horas. Acima de 50 μg/mL, a fluorescência foi mais alta quando comparada a 10 μg/mL, mas não se observou diferenças entre elas ( Figura 2A ).

**Figura 3:**
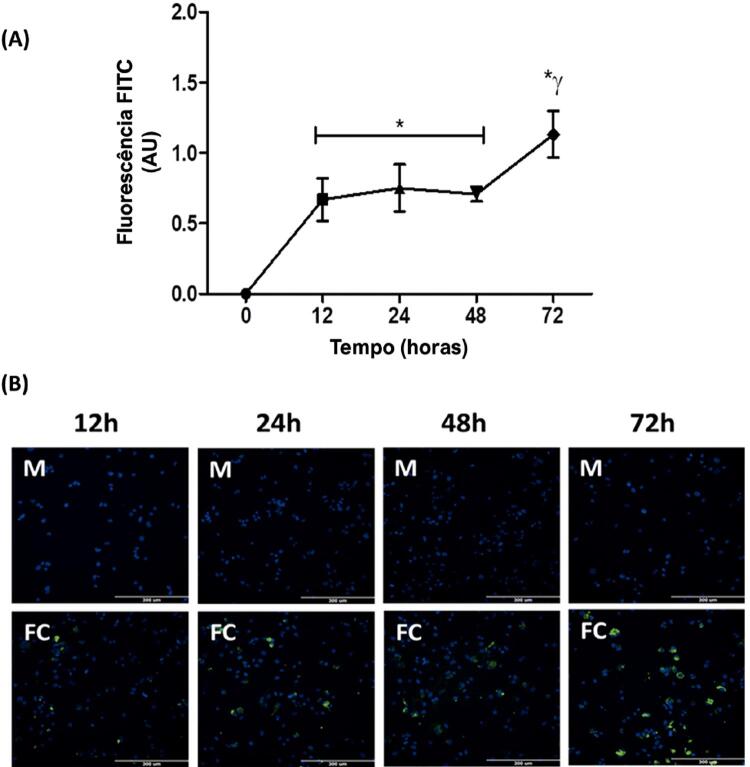
Medição de captação de colesterol aumentada dependente do tempo por macrófagos THP-1. A) Captação de colesterol fluorescente dependente do tempo por macrófagos THP-1 em unidades arbitrárias (UA). B) Imagens representativas de captação de FITC-ox-LDL em macrófagos de THP-1. As THP-1 foram incubadas com 100 ug/mL de FITC-ox-LDL por 0h, 12h, 24h, 48h e 72h. A captação de FITC-ox-LDL foi mostrada em verde e núcleos celulares foram marcados por DAPI (azul). As células foram visualizadas em microscópio de fluorescência (20× objetivas). M, macrófago; FC, células espumosas. Os valores foram expressos como média ± DP. * P <0,05, comparado a células incubadas na ausência de FITC-ox-LDL; y P<0,001, comparado a células incubadas com outras concentrações de FITC-ox-LDL.

Os macrófagos THP-1 foram incubados com 100 μg/mL de FITC-ox-LDL por 0, 12, 24, 48 e 72 h ( Figura 3 ). Observou-se que em 12, 24, 48 e 72 horas, a intensidade da fluorescência das células tratadas com FITC-ox-LDL aumentou significativamente do nível inicial (grupo de controle 0), mas somente após 72 horas a fluorescência foi maior do que a de outros períodos ( Figuras 3A e 3B ).

**Figura 4:**
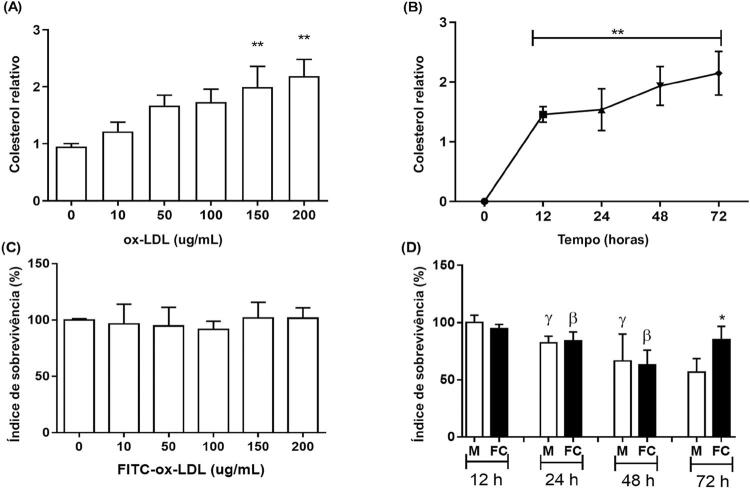
Captação de lipídeos em macrófagos THP-1 e viabilidade das células. A) Captação aumentada dependente da concentração de colesterol por macrófagos THP-1 (Colesterol relativo - o valor foi relativizado para os níveis de proteína celular totais); B) Captação aumentada dependente da concentração de colesterol por macrófagos THP-1 dependente do tempo (Colesterol relativo - o valor foi relativizado para os níveis de proteína celular totais). C) Índice de sobrevivência em concentrações diferentes de FITC-ox-LDL. D) Índice de sobrevivência em tempos de exposição a FITC-ox-LDL diferentes. M, ausência de FITC-ox-LDL no macrófago; FC, células espumosas. Os valores são expressos como média ± DP. P <0,05: * M vs. FC por tempo; ** comparado a células incubadas na ausência de FITC-ox-LDL (tempo 0); γ comparado a M com 12h; β comparado a FC com 12h.

A figura 4 mostra o colesterol relativo, outras técnicas para confirmar a presença de colesterol em dentro das células e a sobrevivência das células nessa condição. Quantitativamente, quando as células foram incubadas por 24 horas em concentrações diferentes, apenas com 150 e 200 µg/mL de ox-LDL, houve um aumento na concentração de colesterol em comparação ao controle não incubado com ox-LDL ( Figura 4A ). Essa condição não causou uma alteração maior na sobrevivência das células ( Figura 4C ). A figura 4B mostra que incubações de 100 µg/mL em tempos de exposição diferentes de ox-LDL não apresentaram diferenças nos períodos diferentes, mas todos os períodos mostraram uma concentração mais alta de colesterol comparadas ao momento 0 ( Figura 4B ). A viabilidade dos macrófagos foi reduzida ao longo do tempo (24, 48 e 72 horas) em comparação com o período de 12 horas, mas as células espumosas apresentaram uma redução na viabilidade apenas nos períodos de 24 e 48 horas, e, em 72 horas, essas células tinham uma viabilidade maior em comparação com os macrófagos em 72 horas, que era semelhante à do grupo das células espumosas no período de 12 horas ( Figura 4D ).

**Figura 5:**
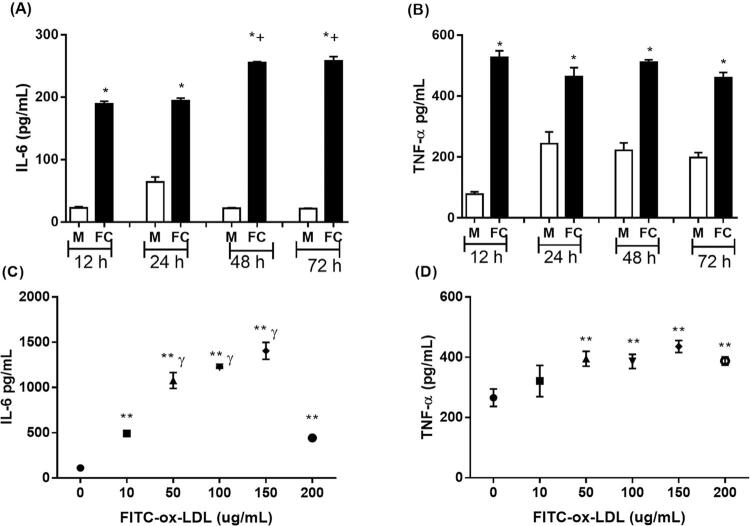
Efeito de tempo e concentração de ox-LDL nas citocinas pró-inflamatórias da célula THP-1: A) Interleucina 6 em tempos diferentes com 100 μg/mL ox-LDL; B) Fator de necrose tumoral alfa (TNF-α) em tempos diferentes com 100 μg/mL ox-LDL. C) Interleucina 6 em concentrações diferentes de ox-LDL tratada por 24 horas. D) Fator de necrose tumoral alfa (TNF-α) em concentrações diferentes de ox-LDL tratado por 24 horas. Os valores são expressos como média ± DP. M, macrófago na ausência de FITC-ox-LDL; FC, células espumosas. * P <0,001, M vs. FC por tempo (Teste T de Student); P <0,05: ** comparado a células incubadas na ausência FITC-ox-LDL (tempo 0); γ comparado com células incubadas de 10 μg/mL. + P < 0.01, comparado com 12h e 24h.

Na produção de citocinas inflamatórias, a curva de tempo mostra que tanto a IL-6 e o TNF-α eram mais altos em células espumosas em comparação a macrófagos em todos os períodos de exposição a ox-LDL ( Figura 5A, 5B ), mas apenas a IL-6 foi mais alta nos períodos de 48 e 72 horas em comparação aos períodos de 12 e 24 horas ( Figura 5A ). Considerando a curva de concentração de ox-LDL, a produção de IL-6 foi mais alta em todas as concentrações testadas, em comparação às células sem exposição a ox-LDL (grupo de controle 0) ( Figura 5C ). Além disso, quando exposta a 50, 100, e 150 µg/mL, a produção de IL-6 foi maior quando comparada à concentração de 10 µg/mL, e a concentração de 200 µg/mL também diminuiu a liberação de IL-6, igualando-se aos valores de concentração de 10 µg/mL. A liberação de TNF-α foi mais expressiva apenas nas concentrações de 50 a 200 µg/mL ( Figura 5D ).

## Discussão

Nossos experimentos documentam uma otimização do método existente de formação de célula espumosa induzida por LDL oxidada para a formação de célula espumosa *in vitro* , a partir de macrófagos THP-1 e da incubação com FITC-ox-LDL, além de verificar a resposta de citocinas tais como a IL-6 e o TNF-α. Com 12 horas de incubação, a formação de célula espumosa acontece com o fenótipo pró-inflamatório M1, ou seja, com um aumento nas concentrações de IL-6 e TNF-α. A caracterização do perfil inflamatório dos macrófagos é importante, pois macrófagos M1 pró-inflamatórios ativados de forma clássica estimulam a aterogênese, enquanto macrófagos M2 estabilizam a placa aterosclerótica.^6^

Outros estudos usaram a técnica de formação de célula espumosa adotando protocolos, como o uso de corante Oil Red O ou LDL marcada com suas próprias sondas, juntamente com LDL oxidada tornada mais complexa com o corante DiL (DiL-ox-LDL). Um estudo conduzido por Xu et al.,^21^ demonstrou que, após a incubação com DiL-ox-LDL (10 µg/mL) por 4 horas, o resultado foi um aumento significativo de captação de ox-LDL em macrófagos; entretanto, esse estudo não avaliou a inflamação nesses macrófagos.^21^ Embora o protocolo de indução de célula espumosa usando-se a sonda DiL-ox-LDL seja mais eficiente em comparação com outras técnicas, ele tem um rendimento menor, ou seja, é necessária uma grande quantidade de materiais para realizá-la, aumentando muito o custo. Além dessa técnica, este trabalho indica que a formação de células espumosas usando-se o corante Oil Red O e incubação de ox-LDL (50 μg/mL) por 24 horas não é um protocolo preciso, já que, nesse protocolo, lipídeos neutros (especialmente triglicérides) são coradas com um corante vermelho-alaranjado,^22^ o que pode levar à baixa especificidade da técnica, já que em células espumosas há mais éster de colesterol e não há lipídeos neutros. Portanto, no presente estudo, os métodos foram otimizados usando-se LDL oxidada marcada com uma sonda fluorescente FITC (FITC-ox-LDL), introduzindo um método prático de coloração para formação de célula espumosa a partir de macrófagos. Usando a marcação de ox-LDL com FITC e a quantificação da inflamação na formação de células, obteve-se um método com a qualidade de sondas fluorescentes de baixo custo, produzindo fotos de alta qualidade.

Para a formação de célula espumosa, a LDL humana foi isolada por ultracentrifugação, oxidada e marcada com sonda de isotiocianato de fluoresceína (FITC). O uso de FITC como sonda fluorescente é amplo, já que o grupo do isotiocianato reage com grupos aminos terminais e primários em proteínas, o que faz dele uma técnica viável e altamente acessível.^11 , 14^ Células THP-1 aderentes acumulam inúmeras gotículas de lipídeos (coloridas em verde) expostas a uma concentração de 100 μg/mL de LDL oxidada por 24 horas, conforme mostrado na literatura.^23 , 24^ Além disso, a THP-1 diferenciada por macrófagos assumiu a aparência morfológica de células espumosas com gotículas de lipídeo fluorescente presentes ao longo do citosol e próximo ao núcleo da maioria das células. Os monócitos de THP-1 foram amplamente usados como modelo de macrófagos *in vitro* , mas pouca atenção foi dedicada à otimização da formação de célula espumosa a partir de macrófagos sem verificar se há inflamação.

A concentração de 100 μg/mL é mais comumente usada na literatura,^23 , 24^ entretanto, os dados do presente estudo mostram que macrófagos derivados de monócitos THP-1 são bem diferenciados em células espumosas com 50 μg/mL FITC-ox-LDL por apenas 12h. Nessas condições, há um acúmulo de colesterol na célula com produção aumentada de citocinas pró-inflamatórias, tais como IL-6 e TNF-α, sem alterar a viabilidade dessa célula. O fenótipo pró-inflamatório é de grande importância para a formação de célula espumosa, já que os componentes presentes na ox-LDL podem induzir diversos efeitos biológicos *in vitro* e *in vivo* , tais como a diferenciação de monócitos, a ativação de células endoteliais, e a ativação do sistema imune. Além disso, há evidências de que sua ação se deve à ativação de TLR4.^25^ Portanto, o processo oxidativo parece estar diretamente envolvido no estímulo dessas substâncias.

Além da concentração de LDL no citoplasma do macrófago, é importante monitorar a produção de citocinas inflamatórias, já que os macrófagos podem contribuir para a aterogênese, especialmente após sua interação com a ox-LDL na camada íntima da artéria, produzindo citocinas e mediadores inflamatórios.^7^ A crescente expressão de marcadores inflamatórios pode ser causada pela ativação de macrófagos durante o processo aterosclerótico, levando a um aumento na captação de ox-LDL.^2^ Os resultados mostrados neste trabalho demonstraram que a produção de IL-6 e TNF-α aumentou em macrófagos quando o tempo de exposição variou. A IL-6 foi liberada em uma concentração mais alta nas células espumosas em comparação com os controles. Além disso, quanto maior o tempo de exposição à ox-LDL, maior a liberação de IL-6, de forma que os períodos de 48 e 72 horas tiveram uma liberação maior em comparação com os períodos de 12 e 24 horas. Considerando o TNF-α, todos os tempos foram maiores do que seus controles, mas não houve diferenças entre os tempos de exposição. Em um ambiente com alta inflamação, é extremamente importante considerar a viabilidade dessas células, pois, nessas condições experimentais, a exposição a FITC-ox-LDL por 72 horas reduziu a viabilidade das células em comparação com a exposição por 12 horas. Juntos, esses dados podem sugerir que a produção de IL-6 e TNF-α poderiam contribuir para a adaptação da fagocitose de macrófagos. Isso é especialmente importante se, nesse microambiente, os macrófagos (M1) estão em maiores quantidades, promovendo um processo inflamatório, induzindo a cronicidade e promovendo efeitos deletérios nos tecidos.

As células espumosas na placa aterosclerótica produzem citocinas pró-inflamatórias que podem contribuir para a inflamação local. Sua natureza inflamatória foi corroborada por estudos *in vitro* que mostram macrófagos M2 derivados de monócitos, que normalmente têm um fenótipo anti-inflamatório, consomem altos níveis de ox-LDL e produzem fatores pró-inflamatórios (IL-6, IL-8, MCP-1) seguidos da formação de célula espumosa, assumindo, dessa forma, um fenótipo pró-inflamatório mais próximo ao M1.^2^ Os macrófagos, *in vivo* , são uma população de células dinâmicas com características fenotípicas e funcionais que são significativamente diferentes entre si, dependendo de seu ambiente de maturação e da natureza de seus estímulos adicionados.^7^ Por exemplo, células THP-1 podem ser direcionadas a um fenótipo M1 usando o IFN-γ,^16^ da forma como usamos em nosso protocolo e que foi confirmada pela alta liberação de citocinas inflamatórias. Outros estudo usam um protocolo de exposição a LDL muito prolongado, com duração de 48 horas ou mais,^26 , 27^ o que mostramos não ter viabilidade, já que, com 12 horas de incubação, a formação da célula espumosa já foi obtida, garantindo um alto grau de inflamação. Além disso, no período de 48 horas, a viabilidade da célula foi reduzida em aproximadamente 50%, prejudicando possíveis intervenções.

Portanto, a falta de um protocolo uniforme que apresenta componentes inflamatórios afeta grandemente a interpretação dos resultados e a capacidade de comparar estudos. Isso se dá porque o desenho do experimento não representa possíveis diferenças fenotípicas e/ou funcionais nas populações de macrófagos que podem ser atribuídas ao uso de protocolos de maturação diferentes, tempo de exposição e concentração de LDL, sem avaliar o perfil inflamatório.

## Conclusão

Os resultados do presente estudo sugerem um modelo que contribui para o entendimento da liberação de IL-6 e TNF-α em resposta a várias concentrações de ox-LDL usando o método otimizado para a formação de células espumosas. Portanto, o entendimento das relações fenotípicas de macrófagos e mecanismos inflamatórios é importante para o desenvolvimento de pesquisas sobre o combate/atenuação da condição de aterosclerose.
