# Supplementary material for: Perfil de IL-6 e TNF na Formação de Células Espumosas: Um Método Aprimorado Usando a Sonda de Isotiocianato de Fluoresceína (FITC)
Source: Arq Bras Cardiol. 2022 Jul 28;119(4):533–41. [Article in Portuguese] doi: 10.36660/abc.20210682 (PMC9563877; doi:10.36660/abc.20210682)
Supplement: Translation

# Translation: Profile of IL-6 and TNF in Foam Cell Formation: An Improved Method Using Fluorescein Isothiocyanate (FITC) Probe

**Authors:** Cynthia Aparecida Castro, Tereza Cristina Buzinari, Rafael Luis Bressani Lino, Heloisa Sobreiro Selistre de Araújo, Fernanda de Freitas Aníbal, Roberto Mario Machado Verzola, Vanderlei Salvador Bagnato, Natalia Mayumi Inada, Gerson Jhonatan Rodrigues

**Affiliations:** 1 Brasil Instituto de Física de São Carlos, Universidade de São Paulo, São Carlos, SP – Brasil; 2 Brasil Departamento de Morfologia e Patologia – Universidade Federal de São Carlos, São Carlos, SP – Brasil; 3 Brasil Departamento de Ciências Fisiológicas – Universidade Federal de São Carlos, São Carlos, SP – Brasil

**Keywords:** Atherosclerosis, Inflammation, Foam Cells, Lipids, Plaque, Atherosclerotic, Isotiocianatos, Fluoresceina

## Abstract

**Background:**

The formation of foam cells occurs due to the increase in low-density plasma lipoprotein (LDL) and dysregulation of inflammation, which is important for the development of atherosclerosis.

**Objective:**

To evaluate the profile of tumor necrosis factor-alpha (TNF-α) and Interleukin-6 (IL-6) in the existing foam cell formation method, optimizing this protocol.

**Methods:**

The LDL was isolated, oxidized, and labeled with a Fluorescein isothiocyanate (FITC) probe. Foam cells were generated from THP-1 human monocyte-derived cells and incubated in the absence (control) or presence of FITC-ox-LDL (10, 50, 100, 150, or 200 μg/mL), for 12, 24, 48, or 72 hours. The accumulated FITC-ox-LDL in the cell was quantified by microscopy. The enzyme-linked immunosorbent assay was evaluated to quantify the IL-6 and TNF-α, with p < 0.05 considered significant.

**Results:**

All the FITC-ox-LDL concentrations tested showed a higher fluorescence when compared to the control, showing a greater accumulation of lipoprotein in cells. The higher the concentration of FITC-ox-LDL, the greater the production of TNF-α and IL-6. The production of IL-6 by foam cells was detected up to the value of 150 µg/mL of the maximum stimulus for LDL. Concentrations above 50 μg/mL LDL stimulated greater release of TNF-α compared to control.

**Conclusions:**

Our model contributes to the understanding of the release of IL-6 and TNF-α in response to different concentrations of ox-LDL, using an optimized method for the formation of foam cells.

## Introduction

Atherosclerosis is one of the most important causes of morbidity and mortality worldwide, and is detected by the accumulation of lipids in macrophages that in this stage are known as foam cells in the sub-endothelial space of the arterial wall.^1^ Foam cell formation occurs by the increase of plasma low-density lipoprotein (LDL), which undergoes various physiological processes mediated by oxidation, acetylation, and denaturation. These modifications are physiological stimuli that favor the Internalization of lipid particles by macrophages generating the foam cell.^2^ Alternative cell types present in the neointima, such as smooth muscle and endothelial cells, can also internalize lipid droplets and transdifferentiate to a state similar to foam cells from macrophages, contributing to the formation of atherosclerotic plaque.^3 , 4^

Macrophages can contribute to the development of atherosclerosis, displaying high heterogeneity^5^ due to its resulting phenotype. This phenotype can be classified as M1 and M2. M1 macrophages are characterized as pro-inflammatory and have a high expression of pro-inflammatory proteins that contribute to the formation of atherosclerotic plaque. M2 macrophages play a preventive role by reducing the size and improving the stability of the plaque, as it has an anti-inflammatory profile.^5 , 6^

Stimulating the pro-inflammatory profile is important in the process of foam cell formation, given that inflammatory mechanisms can act both as precursors in the lipid-centric formation as well as promote atherogenesis via cholesterol absorption and a decrease in cholesterol efflux.^2^ Although hyperlipidemia stimulates atherogenesis by providing more lipids for foam cell formation, some induced inflammatory mediators increase lipid oxidation, such as tumor necrosis factor alpha (TNF-α) and Interleukin-6 (IL-6).^7^ IL-6 is a pleiotropic cytokine that exhibits pro and anti-inflammatory properties, depending on the type of target cell. An increase in IL-6 in atherosclerosis results in effects on multiple cells involved in lipid processing and plaque formation, such as the activation of endothelial cells, smooth muscle cell proliferation, and accumulation of macrophage lipids.^8^ There is now strong evidence for the role of macrophage-derived TNF-α in the development of atherosclerosis and increased vascular inflammation.^9^ Therefore, investigating the physiopathology of foam cell formation is useful in developing new therapeutic interventions for atherosclerosis.^10^

The most commonly used techniques for studying foam cell formation are ox-LDL-labeled quantification inside the macrophages or using non-specific stains such as oil. The present study aimed to evaluate the profile of TNF-α and IL-6 in the existing foam cell formation method, thereby optimizing this protocol. The presence of these inflammatory mediators act as markers of the formation of pro-inflammatory foam cells, the beginning of the formation of atherosclerotic plaque.

## Materials and methods

### Chemicals and reagents

This study used RPMI 1640, Fetal Bovine Serum (FBS) (Vitrocell Embriolife, Campinas, SP, BR), PMSF (phenyl-methyl-sulfonyl-fluoride), Phorbol 12-myristate 13-acetate (PMA), Fluorescein Isothiocyanate (FITC), 4′,6-Diamidine-2′-phenylindole dihydrochloride (DAPI), benzamidine, gentamicin chloramphenicol, aprotinin, Thiazolyl Blue Tetrazolium Bromide (MTT), which were purchased from Sigma-Aldrich, St. Louis, MO, USA, Amplex Red Cholesterol Assay Kit (Catalog no. A12216, Invitrogen, Molecular Probes, Eugene, OR); IL-6 and TNF-α R&D Systems, 614 McKinley Pl NE, Minneapolis, MN, USA.

### LDL isolation

The present study was approved by the Human Research Ethics Committee at the Universidade Federal de São Carlos - UFSCar (#2.243.706) and the participants provided their written consent. Blood was collected from 10 normolipidemic volunteers (men and women, aged 18 to 45 years), and plasma was obtained after centrifugation at 1,000 g for 15 min in the presence of K_2_EDTA 0.1mL for each 5 mL of blood. Next, benzamidine (2 mM), gentamicin (0.5%), chloramphenicol (0.25%), PMSF (phenyl-methyl-sulfonyl-fluoride) (0.5 mM), and aprotinin (5µl/mL) (all acquired from Sigma-Aldrich, St. Louis, MO, USA) were added to the plasma pool, as described in previous report.^11^ The plasma density was raised to 1.021 g/mL by KBr (the plasma volume is multiplied by factor 0.3265, and the amount is then obtained in grams of solid KBr). After, 2.5 mL of plasma was added to the polypropylene tube (4 mL), and the tube was completed with a KBr solution of d = 1.006. The LDL was isolated by ultracentrifugation (337 g for 4h at 4°C) in a SW60TI fixed-angle rotor (Beckman Coulter, Beckman). The yellow-orange LDL fraction remained in the infranatant. The LDL fraction was collected by suction, using a 1 mL syringe. The collected LDL was dialyzed in the dark at 4ºC in 2 L of PBS, pH 7.4, with four PBS exchanges for 24 hours. After dialysis, LDL was filtered (0.22 μm) and stored at 4°C. The protein concentration was determined by using the Folin phenol reagent method.^12^

### Oxidative modification of LDL

Oxidized LDL (ox-LDL) was obtained by incubating LDL with CuSO4 (5μmol/mL per mg of LDL protein/ 4 h/ 37°C). Oxidation was stopped by adding 20 µmol/mL EDTA. The degree of oxidation was determined by measuring the ferrous oxidation-xylenol orange.^13^ After oxidation, the ox-LDL was dialyzed in the dark for 24 h at 4°C and washed 4 times with 2 L of PBS and EDTA (0.3 mM).

### Fluorescent labeling of LDL

The oxidized LDL was labeled with Fluorescein isothiocyanate (FITC). All procedures were performed in the dark. LDL (1 mg/mL) and FITC (50 μg /mL) were mixed and incubated at 37°C for 3h. Unbound FITC was removed by dialysis against PBS for 48h at 4°C with eight changes of PBS and filtered through a 0.22 μm filter.^14^ FITC-ox-LDL was then stored at 4°C and used for up to two months.

### Cell culture

The THP-1 human monocyte-derived cell line was purchased from the *Rio de Janeiro Cell Bank,* Rio de Janeiro, *Brazil,* and was grown at 37°C in a 5% CO_2_atmosphere to a density of 10^6^cells/mL. The growth medium for the THP-1 cells was RPMI Medium 1640 supplemented with 10% Fetal Bovine Serum (FBS) (Gibco BRL), 50 mg/L Gentamicin Sulfate, and 2 mg/L Amphotericin B. The THP-1 cells were used in the experiments for induction in macrophages, using the 100 nM phorbol myristate acetate (PMA, Sigma)^15^ and interferon (IFN)-γ (500 U/mL) to induce the M1 phenotype.^16^ PMA induces THP-1 cell differentiation through direct interaction with PKCδ, which binds to Thrombomodulin and activates ERK1/2, which in turn increases cell the cycle inhibitor p21^Cip1^expression via NF-kB p65 signaling. In addition, ERK1/2 participates in the phosphorylation of paxillin, cofilin, LIMK1, and PYK2, which mediate cytoskeletal remodeling to promote differentiation.^17^ Interferon γ (IFN-γ), through the activator of transcription 1 (STAT1), favors the polarization of M1 macrophages, which produce pro-inflammatory mediators, including TNF-α, IL-6, and IL-1.^18^ After this induction, the THP-1 macrophage cells were incubated without FITC-ox-LDL or with 10, 50, 100, 150, or 200 μg/mL, for different times (12, 24, 48 or 72 hours), depending on the experimental purpose.

### Cellular uptake of cholesterol

To induce THP-1 monocyte differentiation in macrophages, THP-1 monocytes (10^4^cells/mL) in 96-well plates were treated with 100 nM PMA for 48 hours at 37°C. To identify the best ox-LDL concentration to induce foam cell formation, a concentration-response curve was performed: for 24 hours at 10 μg/mL, 50 μg/mL, 100 μg/mL, 150 μg/mL, and 200 μg/mL FITC-ox-LDL + IFN-γ (500 U/mL). For the temporal analysis, differentiated cells were incubated in the absence or presence of FITC-ox-LDL (100 μg/mL) + interferon γ (500 U/mL) for 12h, 24h, 48h, and 72h. The cell nucleus was labeled with 1 μg/mL DAPI fluorescent probe (Sigma) for 10 minutes and washed 3 times with PBS. To analyze the fluorescence image, an automated fluorescence microscope system, ImageXpress Micro (Molecular Devices), with 495nm excitation, 525 nm FITC-ox-LDL emission, 340 nm excitation, and 488 nm emission for DAPI was used.

### Cholesterol/cholesteryl ester quantitation in cell lysate

Foam cell cholesteryl ester content was quantified by the Amplex Red Cholesterol Assay Kit (Catalog no. A12216, Molecular Probes, Eugene, OR), according to the manufacturer’s protocol. For this analysis, the THP-1 cells (2x10^6^cells/well) were cultured in 6-well plates; differentiated into macrophages, as described above; and incubated with or without ox-LDL. Foam cells were fixed in 2% paraformaldehyde for 15 min, washed once with PBS, and incubated with a 200 μl/well of absolute ethanol for 30 min at 4°C to extract cellular lipids. Cholesterol content was determined by incubating 50 µl of ethanol-extracted lipids diluted in 1x reaction buffer (0.1 M K_2_HPO_4_, pH 7.4, 0.05 M NaCl, 5 mM Cholic acid, 0.1% Triton X-100) or undiluted solution with 50 µl of assay solution (total cholesterol) or 50 µl of assay solution lacking cholesterol esterase (free cholesterol), for 30 min at 37°C in the dark and then by measuring the fluorescence (HTS-7000 microplate fluorometer; 530-nm excitation, 590-nm). The value was relativized for the total cellular protein levels. To quantify the total cellular protein levels, the lipid-extracted foam cells were incubated with 0.1% (weight/volume ratio [w/v]) SDS/0.2 M NaOH for 30 min at room temperature to extract the cellular protein. Total cellular protein levels were determined by using the Folin phenol reagent method.^12^ Total cholesterol and cholesteryl ester levels were represented as nanograms of total cholesterol or cholesteryl ester per microgram of protein.

### Cytokine measurements

Quantification of inflammatory cytokines in foam cell lysate was performed using the enzyme-linked immunosorbent assay (ELISA). IL-6 and TNF-α concentration in the supernatants of macrophages were measured using DuoSet kits (R&D Systems, 614 McKinley Pl NE, Minneapolis, MN, USA). Macrophages that incubated in absence of ox-LDL were defined as the control group (M).

### Cell viability

Cell viability was determined by MTT (Thiazolyl Blue Tetrazolium Bromide) (Sigma- Aldrich, St. Louis, MO, USA) (Mosmann, 1983). THP-1 monocytes (10^4^cells/mL) were seeded in 96-well plates and treated with 100 nM PMA for macrophage differentiation, for 48 hours, maintained at 37°C in a humidified incubator containing 5% CO_2_. After 48 hours, cells were exposed for 24 hours to 10 μg/mL, 50 μg/mL, 100 μg/mL, 150 μg/mL, and 200 μg/mL of FITC-ox-LDL + interferon γ (500 U/mL). The analysis of cell viability over time was also performed, where the cells were incubated in the absence (Control group - M) or presence of FITC-ox-LDL (FC) (100 μg/mL) + interferon γ (500 U/mL) for 12h, 24h, 48h, and 72h. Later, 5 mg/mL of MTT was added, followed by 4 hours of incubation at 5% CO_2,_37°C. After this time, 100 μL of dimethylsulfoxide (DMSO) was added, and the plate remained on the plate shaker for 10 minutes. The absorbance was measured at 540 nm, using a microplate reader SpectraMax GeminiXS (Molecular Devices, Sunnyvale, CA, USA).

### Statistical analysis

The entire study was carried out at least in triplicate, in three independent experiments, according to recommendations for Good Cell Culture Practice (GCCP).^19 , 20^ Data normality was verified by the Kolmogorov-Smirnov test; equality of variance (Levene’s test). All values were presented as mean ± standard deviation (SD). To determine the difference between conditions, ANOVA was applied with the Bonferroni post hoc test for multiple comparisons. To determine the difference between two conditions, the unpaired Student’s t-test was used (SigmaStat version 3.5; Systat). The significance level adopted in the statistical analysis was 5%.

## Results

There was a greater accumulation of ox-LDL labeled with a FITC probe (indicated by the presence of green fluorescence), not only in the perinuclear area, but also distributed throughout the cytosol of most cells, as shown in Figure 1B . As expected, no accumulation of LDL was found in the untreated cells ( Figure 1A ), which exhibited only blue fluorescence, which labels the cell nucleus.

**Figure 1:** Representative images of FITC-ox-LDL uptake in THP-1 macrophages. THP-1 were incubated in the absence (Ctrl group - A or presence B) of indicated concentrations of FITC-ox-LDL for 24h. Cells were then washed, fixed, and examined, using a 546 nm filter set. FITC-ox-LDL uptake was shown in green and the cells´ nucleus was labeled using DAPI (blue).

In Figure 2B, the microscopic fluorescence images show that FITC-ox-LDL was absorbed in all concentrations, showing higher fluorescence when compared to the control, using 24h of incubation. Above 50 μg/mL, the fluorescence was higher when compared to 10 μg/mL, but no difference was observed between them ( Figure 2A ).

**Figure 2:** Measurement of FITC-ox-LDL concentration in THP-1 cells. A) Concentration-dependent fluorescent cholesterol uptake by THP-1 macrophages in arbitrary unit (AU). B) Representative images of FITC-ox-LDL uptake in THP-1 macrophages. THP-1 were incubated in the absence (Control group: 0) or presence of indicated concentrations of FITC-ox-LDL (10 – 200 μg/mL) for 24 h. FITC-ox-LDL uptake was shown in green, and cell nucleus was labeled using DAPI (blue). Cells were viewed under fluorescence microscope (20× objectives). Values are expressed as mean ± SD. * P < 0.05, compared to cells incubated with 10 μg/mL; ** P < 0.01, compared to cells incubated in absence of FITC-ox-LDL.

The THP-1 macrophages were incubated with 100 μg / mL of FITC-ox-LDL for 0, 12, 24, 48, and 72 h ( Figure 3 ). It was observed that within 12, 24, 48, and 72h the fluorescence intensity of the cells treated with FITC-ox-LDL increased significantly from the background level (Control group 0), but only after 72h was the fluorescence greater than other times ( Figure 3A and 3B ).

**Figure 3:** Measurement of time-dependent increased uptake of cholesterol by THP-1 macrophages. A) Time-dependent fluorescent cholesterol uptake by THP-1 macrophages in arbitrary unit (AU). B) Representative images of FITC-ox-LDL uptake in THP-1 macrophages. THP-1 were incubated which 100 ug/mL of FITC-ox-LDL for 0h, 12h, 24h, 48h, and 72h. FITC-ox-LDL uptake was shown in green, and cell nucleus was labeled by DAPI (blue). Cells were viewed under fluorescence microscope (20× objectives). M, macrophage; FC, foam cells. Values are expressed as mean ± SD. * P < 0.05, compared to cells incubated in absence of FITC-ox-LDL; γ P < 0.001, compared to cells incubated with the other FITC-ox-LDL concentrations.

Figure 4 showed the relative cholesterol, other techniques to confirm the presence of cholesterol in cells, and the survival of the cells in this condition. Quantitatively, when the cell was incubated for 24h in different concentrations, only with 150 and 200 µg/mL of ox-LDL, a greater cholesterol concentration was found when compared to the control that was not incubated with ox-LDL ( Figure 4A ). This condition did not cause a major change in cell survival ( Figure 4C ). In Figure 4B , incubations of 100 µg/mL at different times of exposure of ox-LDL showed no difference between times, but all times showed a greater concentration of cholesterol the compared to time 0 ( Figure 4B ). Macrophages reduced their viability over time (24, 48, and 72h) as compared to the time of 12h; however, foam cells showed a reduction in viability only at times of 24 and 48h. By contrast, in 72h, these cells had a greater viability when compared to macrophages in 72h, which was similar to that found for the group of foam cells at 12h ( Figure 4D ).

**Figure 4:** Lipid uptake in THP-1 macrophage and cell viability. A) Concentration-dependent increased uptake of cholesterol by THP-1 macrophages (Relative cholesterol - the value was relativized for the total cellular protein levels); B) Time-dependent increased uptake of cholesterol by THP-1 macrophages (Relative cholesterol - the value was relativized for the total cellular protein levels). C) Survival rate in different concentrations of FITC-ox-LDL. D) Survival rate in different exposure time of FITC-ox-LDL. M, macrophage absence of FITC-ox-LDL; FC, foam cells. Values are expressed as mean ± SD. P < 0.05: * M vs FC per Time; ** compared to cells incubated in absence of FITC-ox-LDL (time 0); γ compared to 12h M; β compared to 12h FC.

In the production of inflammatory cytokines, the time curve shows that both IL-6 and TNF-α were higher in foam cells when compared to macrophages at each time of exposure to ox-LDL ( Figure 5A, 5B ), but only IL-6 was higher in times 48h and 72h when compared to 12h and 24h ( Figure 5A ). Considering the ox-LDL concentration curve, IL-6 production was higher in all tested concentrations when compared to cells without exposure to ox-LDL (control group 0) ( Figure 5C ). In addition, when exposed to 50, 100, and 150 µg/mL, the production of IL-6 was greater when compared to the concentration of 10 µg/mL. The concentration of 200 µg/mL also decreased the release of IL-6, matching the values of concentration of 10 µg/mL. The release of TNF-α was only more expressive at concentrations of 50 to 200 µg / mL ( Figure 5D ).

**Figure 5:** Time and concentration effect of ox-LDL in the proinflammatory cytokines of THP-1 cell: A) Interleukin 6 at different times with 100 μg/mL ox-LDL; B) Tumor Necrosis Factor alpha (TNF-α) at different times with 100 μg/mL ox-LDL. C) Interleukin 6 in different concentrations of ox-LDL, treated for 24 hours. D) Tumor Necrosis Factor alpha (TNF-α) in different concentrations of ox-LDL, treated for 24 hours. Values are expressed as mean ± SD. M, macrophage absence of FITC-ox-LDL; FC, foam cells. * P < 0.001, M vs FC per Time (Test T Student); P < 0.05: ** compared to cells incubated in absence of FITC-ox-LDL (time 0); γ compared to cells incubated with 10 μg/mL. + P < 0.01, compared to 12h and 24h.

## Discussion

Our experiments document an optimization of the existing method of oxidized LDL-induced foam cell formation for *in vitro* foam cell formation, from THP-1 macrophages and incubation with FITC-ox-LDL, in addition to the verification of the response of such cytokines as IL6 and TNF-α. With 12h of incubation, foam cell formation takes place with the M1 pro-inflammatory phenotype, that is, with an increase in the concentrations of Il-6 and TNF-α. The characterization of the inflammatory profile of macrophages is important, considering that classically activated pro-inflammatory M1 macrophages stimulate atherogenesis, while M2 macrophages stabilize the atherosclerotic plaque.^6^

Other studies have used the foam cell formation technique, adopting such protocols as Oil Red O staining or labeled LDL with their own probes, together with oxidized LDL complexed with DiL dye (DiL-Ox-LDL). A study conducted by Xu et al.,^21^ showed that incubation with DiL-ox-LDL (10 µg/mL) for 4h resulted in a significant increase in ox-LDL uptake in macrophages; however, they did not evaluate the inflammation of these macrophages.^21^ Although the foam cell induction protocol using the DiL-ox-LDL probe is more efficient when compared to other techniques, it also has a low yield, that is, a large amount of material is needed to perform it, making it rather costly. In addition to this technique, this work points out that foam cell formation using Oil Red staining and LDL-ox incubation (50 μg/mL) for 24h is not an accurate protocol, since in this protocol neutral lipids (mainly triglycerides) are stained with an orange-red dye,^22^ which may cause a low specificity in the technique, as in foam cells there is more cholesterol ester and no neutral lipids. Therefore, the present study sought to optimize the methods using oxidized LDL labeled with an FITC fluorescent probe (FITC-ox-LDL), introducing a simple and practical staining method for foam cell formation from macrophages. Using ox-LDL labeling with FITC and the quantification of inflammation in cell formation, a method with the quality of low-cost fluorescent probes was obtained, producing high quality photos.

For foam cell formation, human LDL was isolated by ultracentrifugation, oxidized, and labeled with fluorescein isothiocyanate conjugate (FITC). The use of FITC as a fluorescent probe is widely used because the isothiocyanate group reacts with terminal and primary amino groups in proteins, making it a viable and highly accessible technique.^11 , 14^ Adherent THP-1 cells accumulate numerous lipid droplets (stained with green) upon exposure to a 100 μg/mL concentration of oxidized LDL for 24 hours, as shown in the literature.^23 , 24^ In addition, the macrophage-differentiated THP-1 assumed the morphological appearance of foam cells with fluorescent lipid droplets present along the cytosol and near to the nucleus of most cells. THP-1 monocytes have been extensively used as an *in vitro* model of macrophages, but little care has been given to optimizing foam cell formation from macrophages without checking for inflammation.

The concentration of 100 μg/mL is most commonly used in the literature;^23 , 24^ however, the present study’s data show that macrophages derived from THP-1 monocytes are well differentiated in foam cells with 50 μg/mL FITC-ox-LDL for only 12h. Under these conditions, there is an accumulation of cholesterol in the cell with an increased production of pro-inflammatory cytokine drugs, such as IL-6 and TNF-α, without altering the viability of this cell. The pro-inflammatory phenotype is of great importance in foam cell formation, as the components present in ox-LDL can induce diverse biological effects in vitro and *in vivo* , such as monocyte differentiation, activation of endothelial cells, and activation of the immune system. Moreover, there is evidence that its action is due to the activation of TLR4.^25^ Therefore, the oxidative process seems to be directly involved in the stimulation of these substances.

In addition to the concentration of LDL in the macrophage cytoplasm, it is important to monitor the production of inflammatory cytokines, as macrophages can contribute to atherogenesis, mainly after its interaction with ox-LDL in the intima layer of the artery, producing cytokines and inflammatory mediators.^7^ The increasing expression of inflammatory markers can be caused by the activation of macrophages during the atherosclerotic process, leading to an increase in the uptake of ox-LDL.^2^ The results shown in this work demonstrated that IL-6 and TNF-α production increased in macrophages when exposed to different exposure times. IL-6 was released in a higher concentration in the foam cells when compared to its controls; in addition, the longer the exposure time to the ox-LDL, the greater the release of IL-6, so that 48h and 72h had a greater release when compared to 12h and 24h. Considering TNF-α, all times were greater than their controls, but there was no difference between exposure times. In an environment with high inflammation, it is of utmost importance to consider the viability of these cells, so that under these experimental conditions, the 72-hour exposure to FITC-ox-LDL reduced cell viability when compared to 12 hours of exposure. Together, these data may suggest that the production of IL-6 and TNF-α could contribute to the upregulation of macrophage phagocytosis, especially if in this microenvironment the macrophages (M1) are in greater quantities, thus promoting an inflammatory process, inducing chronicity, and promoting deleterious effects on tissues.

The foam cells in the atherosclerotic plaque produce proinflammatory cytokines that may contribute to local inflammation. Their inflammatory nature has been supported by *in vitro* studies that show human monocyte-derived M2 macrophages, which normally have an anti-inflammatory phenotype, consume high levels of ox-LDL, and produce proinflammatory factors (IL-6, IL-8, MCP-1), followed by the formation of foam cell, thus taking on a more M1-like proinflammatory phenotype.^2^ Macrophages, *in vivo* , are a dynamic cell population with both phenotypic and functional traits that differ significantly one with another, depending on their maturation environment and the nature of the added stimuli.^7^ For example, THP-1 cells can be directed to an M1 phenotype using the IFN-γ,^16^ as we used in our protocol and which was confirmed by the high release of inflammatory cytokines. Other studies use a very prolonged LDL exposure protocol lasting 48h or more,^26 , 27^ which we show has no viability, given that, with 12h of incubation, the foam cell formation is already obtained, ensuring a high degree of inflammation. In addition, at 48h, the cell viability was reduced by approximately 50%, hindering possible interventions.

Thus, the lack of a uniform protocol that presents inflammatory components greatly affects the interpretation of results and the ability to compare studies. This is because the experimental design does not represent possible phenotypic and/or functional differences in the macrophage populations that are attributable to the use of different maturation protocols, exposure time, and LDL concentration, without evaluating the inflammatory profile.

## Conclusion

The present study’s results suggest a model that contributes to the understanding of the release of IL-6 and TNF-α in response to different concentrations of ox-LDL using an optimized method for the formation of foam cells. Therefore, the understanding of the phenotypic relationships of macrophages and inflammatory mechanisms is important for the development of research to fight/attenuate the condition of atherosclerosis.
